# Reversing Platinum Resistance in High-Grade Serous Ovarian Carcinoma: Targeting BRCA and the Homologous Recombination System

**DOI:** 10.3389/fonc.2014.00034

**Published:** 2014-03-03

**Authors:** W. Ruprecht Wiedemeyer, Jessica A. Beach, Beth Y. Karlan

**Affiliations:** ^1^Women’s Cancer Program, Samuel Oschin Comprehensive Cancer Institute, Cedars-Sinai Medical Center, Los Angeles, CA, USA; ^2^Department of Obstetrics and Gynecology, David Geffen School of Medicine, University of California Los Angeles, Los Angeles, CA, USA; ^3^Graduate Program in Biomedical Sciences and Translational Medicine, Cedars-Sinai Medical Center, Los Angeles, CA, USA

**Keywords:** high-grade serous ovarian cancer, platinum resistance, BRCA1, BRCA2, cyclin-dependent kinases, cyclin E1, cyclin-dependent kinase inhibitors, homologous recombination

## Abstract

Resistance to platinum chemotherapy is one of the main factors driving ovarian cancer mortality, and overcoming platinum resistance is considered one of the greatest challenges in ovarian cancer research. Genetic and functional evidence points to the homologous recombination (HR) DNA repair system, and BRCA1 and BRCA2 in particular, as main determinants of response to platinum therapy. *BRCA*-mutant ovarian cancers are especially sensitive to platinum, associated with better survival, and amenable to poly ADP ribose polymerase inhibitor treatment. Here, we discuss a therapeutic concept that seeks to disrupt HR capacity via targeting of BRCA1 and BRCA2 functionality in order to reverse platinum resistance in BRCA-proficient high-grade serous ovarian cancers (HGSOC). We review the molecular signaling pathways that converge on BRCA1 and BRCA2, their activation status in ovarian cancer, and therapeutic options to modulate BRCA function. Several recent publications demonstrate efficient chemosensitization of BRCA-proficient cancers by combining targeted therapy with standard platinum-based agents. Due to its inherent genomic heterogeneity, molecularly defined subgroups of HGSOC may require different approaches. We seek to provide an overview of available agents and their potential use to reverse platinum resistance by inhibiting the HR system, either directly or indirectly, by targeting oncogenic activators of HR.

## Introduction

Platinum-based chemotherapy agents, such as cisplatin, have been used in the treatment of ovarian carcinoma since the late 1970s. Cisplatin significantly improved the overall survival (OS) of women with ovarian cancer, leading to its adoption as the backbone of most chemotherapeutic regimens (1, 2). Carboplatin, a cisplatin analog, with an improved toxicity profile and equivalent therapeutic efficacy has replaced cisplatin as a standard of care since the mid-1980s (3). The next major advance in chemotherapy for epithelial ovarian cancer occurred with the introduction of the mitotic inhibitor paclitaxel, which further improved OS when combined with platinum (4, 5). Despite these advances, tumor recurrences still occur in the majority of ovarian cancer patients and cures remain too infrequent.

Epithelial ovarian cancer is a heterogeneous disease with multiple histological subtypes and multiple subclones even within a given patient’s tumor. High-grade serous ovarian cancer (HGSOC) accounts for the majority of epithelial ovarian cancers (68%). A recent comprehensive analysis by The Cancer Genome Atlas (TCGA) revealed HGSOC to be highly genomically unstable with *TP53* gene mutations in more than 96% of cases, and less frequent mutations in *BRCA1* and *BRCA2*, while mutation frequencies for all other genes are each <5% (6). Other ovarian cancer subtypes, such as clear cell and endometrioid ovarian cancers have fewer *TP53* mutations and are not commonly associated with *BRCA* gene mutation (7, 8). In addition, TCGA identified a plethora of recurrent DNA copy number changes affecting known oncogenes and tumors suppressor genes. The genomic complexity of HGSOC may explain previous failures of targeted therapy approaches in unselected patient populations. HGSOC has a poor prognosis likely due to a combination of factors, including late stage at presentation and the development of chemoresistance (9). Most patients with advanced (stage III and IV) HGSOC undergo cytoreductive surgery followed by combination platinum- and taxane-based chemotherapy (4, 5). While initial response rates are quite high (~80%), the majority of patients ultimately relapse due to the emergence of chemoresistant disease (10). Once patients develop resistant disease, the options for effective salvage treatment are limited. Clinical trials investigating the inclusion of alternative chemotherapeutic and biologic agents in recurrent platinum-resistant ovarian cancer have failed to demonstrate significant improvements in OS (11), and the 5-year survival rate has remained relatively unchanged at 43% for several decades (12). Thus, there is a critical need to identify and understand the molecular mechanisms and biological pathways that contribute to platinum resistance in HGSOC.

Upon entering a cell, platinum-based compounds generate inter- and intra-strand DNA adducts that activate the DNA damage response (DDR) and subsequently induce DNA repair (13). In the absence of a functional DNA repair system, damage accumulates and cell death ensues. Here, we discuss a therapeutic concept that seeks to reverse platinum resistance in HGSOC via targeting the DNA homologous recombination (HR) repair pathway and the *BRCA1* and *BRCA2* genes in particular. We will review the role of BRCA1 and BRCA2 in determining the platinum response of the cell as well as the concept of synthetic lethality that has led the introduction of poly ADP ribose polymerase (PARP) inhibitors for the treatment of *BRCA*-mutant HGSOC. We will then outline pharmacological strategies to mimic “BRCAness” in BRCA-wildtype HGSOC and explore the use of molecularly targeted agents to exploit this pathway and sensitize the cell to platinum-induced lethality.

## DNA Repair Pathways and Platinum Resistance

Platinum resistance is a complex phenotype characterized by decreased platinum uptake, increased metabolic turnover, inhibition of pro-apoptotic signals, and restored DNA repair capacity [reviewed in Ref. (14, 15)]. Due to this complexity, the development of chemoresistant disease is assumed to be a dynamic process involving multiple mechanisms. As DNA alkylating agents, the cytotoxic effects of platinum drugs are largely dependent on the cell’s ability to detect and repair DNA damage. Several DNA repair pathways exist and have been linked to platinum resistance. Nucleotide excision repair (NER) is the primary pathway used for intrastrand platinum adduct removal and is an important mediator of responsiveness to platinum-based chemotherapy (16). High NER activity is correlated with platinum resistance (17, 18). The mismatch repair (MMR) system functions to repair single-base pair mismatches and erroneous insertions and deletions that occur during DNA replication and recombination. Mutation and decreased expression of MMR components, MLH1 and MSH2, have been documented in ovarian and other cancers, and correlated with prognostic indicators including chemotherapy response (19, 20). In ovarian cancer, deficiencies in MMR and subsequent microsatellite instability (MSI) are estimated to account for tumor development in <10% of cases (20). However, the role of MMR inactivation and MSI in platinum response in HGSOC remains controversial, as several studies have reached conflicting conclusions (21, 22).

The most lethal lesions induced by platinum agents are DNA double strand breaks (DSBs), which are a result of platinum-induced interstrand crosslinks. These DSBs are particularly toxic as both strands of DNA are affected and there is no intact complimentary strand to utilize as a template for repair. DSBs are repaired by two major pathways within the cell: non-homologous end-joining (NHEJ) and HR. The preferred method of DSB repair is HR, as NHEJ is inherently mutagenic and can result in undesirable insertions and/or deletions. HR is a highly conserved pathway that provides error-free repair of DSBs by using the intact sister chromatid as a template, which fixes the break while maintaining sequence integrity. Due to this requirement, HR occurs during G_2_ and S phases of the cell cycle (23). Two of the most well-known HR proteins are BRCA1 and BRCA2. BRCA1 is a multipurpose protein that participates in DDR activation, cell cycle checkpoint initiation, and DSB repair as a component of several supercomplexes (24). In HR, BRCA1 is localized to DSBs through its association with the abraxas-RAP80 complex, and promotes 5′-end resection of the break in cooperation with other proteins (25, 26). BRCA1 is also required in the later stages of HR, where its interaction with PALB2 and BRCA2 is necessary for the recruitment of RAD51 to DSBs and subsequent strand invasion of the sister chromatid for DNA repair (27, 28). Of note, the only well-documented function of BRCA2 is its direct binding of RAD51 in HR (29).

## The BRCA Paradox

Mutations in *BRCA1* or *BRCA2* are found in the majority of hereditary breast and ovarian cancers and greatly increase lifetime risk for both cancers. Moreover, somatic mutations in at least one of the *BRCA* genes are present in a significant proportion of sporadic HGSOC, rendering *BRCA1* and *BRCA2* as two of the most frequently mutated tumor suppressor genes that guard against the transformation of serous epithelium to HGSOC. However, once an advanced tumor has developed, *BRCA*-mutant HGSOC are associated with better survival than wildtype HGSOC. This seeming paradox was first described by comparing outcomes of women with hereditary epithelial ovarian cancer to those of women with sporadic ovarian cancer. *BRCA* mutation carriers had significantly prolonged survival compared to patients with sporadic disease (30, 31). A meta-analysis of 26 studies comparing 1213 cases with germline *BRCA* mutations and 2666 non-carriers determined that the 5-year survival rate was 36% for non-carriers, 44% for *BRCA1* mutation carriers, and 52% for *BRCA2* mutation carriers (32). Further, the analysis by TCGA of 316 HGSOC confirmed that *BRCA*-mutant HGSOC (both hereditary and sporadic) are associated with better survival than *BRCA*-wildtype HGSOC (6). Collectively, these studies suggest that *BRCA*-deficient HGSOC respond better to standard therapies, specifically platinum chemotherapy, compared to *BRCA*-wildtype cancers, and further, that an intact HR system seems to be crucial for the survival of platinum-treated ovarian cancer cells.

### Restored BRCA function in platinum-resistant cancers

An independent line of evidence supporting intact BRCA and HR function as one of the main determinants of chemosensitivity emerged from the analysis of platinum-resistant cells in which BRCA function had been restored by secondary mutations. Sakai et al. analyzed cisplatin-resistant subclones of the CAPAN1 pancreatic cancer cell line, which carries a 6174delT frame-shift mutation and lacks wildtype BRCA2 (33). Fifty percent (7/14) of the resistant clones had restored expression of *BRCA2* by intragenic deletions, insertions, or deletions/insertions. In all clones, the reading frame had been restored, and a functional protein was expressed. Similarly, frame-shift mutations in the *BRCA1* gene can be reversed by secondary mutations in cisplatin-resistant ovarian cancers (34, 35). Mechanistically, secondary mutations could be the result of error-prone DNA repair in cells that lack a functional HR system. In the presence of cisplatin, cancer cells with restored HR function are expected to have a strong selection advantage and may thus become the dominant cell clone in recurrent cancers. In a mouse model of mammary tumorigenesis induced by combined loss of *Brca1* and *p53* (K14-Cre; Brca1^flox/flox^; p53^flox/flox^), *Brca1*-null tumors do not become cisplatin-resistant over the course of at least six cycles of cisplatin treatment (36). In contrast to point mutations or small insertions/deletions found in human cancers, large genetic deletions resulting from Cre-mediated recombination in this mouse model are irreversible. The inability of these murine cancer cells to restore functional *Brca1* expression may explain their sustained platinum sensitivity. Interestingly, platinum treatment cannot fully eradicate the breast tumors in this model, leaving a small fraction of surviving cells that can repopulate the tumor following withdrawal of cisplatin (36). It is tempting to speculate that the few surviving clones escape from platinum-induced death by employing mechanisms related to reduced proliferation, such as acquisition of cancer stem cell properties, or complete exit from the cell cycle [dormancy, reviewed in Ref. (37)].

### Exploiting loss of BRCA function in a synthetic lethal approach using PARP inhibitors

Synthetic lethality is defined as death resulting from concomitant mutation of two genes if mutation of either gene alone is associated with viability but mutation of both is lethal (38). This concept can be expanded to more than two genes and to pharmacologically modulated gene activity, e.g., loss-of-function following pharmacological inhibition of protein that is critically required in cancer cells. In the context of anticancer therapy, a synthetic lethal approach may take advantage of somatic mutations that render the tumor sensitive to specific chemotherapeutic agents but spare normal cells without the mutation. Alternatively, tumor-specific dependency on individual genes or signaling pathways (“oncogene addiction”) can expose synthetic lethal vulnerabilities.

In ovarian cancer, the most prominent example of synthetic lethality involves PARP inhibition in *BRCA*-mutant cancers. PARP is a DNA repair enzyme and part of the base excision repair (BER) pathway. The HR defect in *BRCA*-mutant cancers renders them particularly sensitive to inhibition of other DNA repair pathways that compensate for loss of HR activity. Concomitant defects in the HR and BER pathways are synthetic lethal; DNA damage accumulates in PARP inhibitor-treated *BRCA*-mutant cells and may be repaired by error-prone mechanisms, such as NHEJ. As a result, complex chromatid rearrangements ensue that lead to G_2_/M phase cell cycle arrest and subsequent cell death (39).

Based on the concept of synthetic lethality, several PARP inhibitors (PARPi), such as Olaparib, have entered clinical trials for ovarian cancer and other *BRCA*-associated cancers. Ovarian cancer-specific trials in patients with recurrent *BRCA*-mutant cancers showed high response rates between 30 and 60% for Olaparib (40, 41) and increased progression-free survival (42). While *BRCA*-mutant cancers are especially sensitive to PARPi, a significant proportion of BRCA-proficient HGSOC are thought to exhibit a “BRCAness” phenotype, which is caused by HR defects other than *BRCA* mutation (43). HGSOC with the BRCAness phenotype are also predicted to be sensitive to PARPi, and the identification of these cancers within the pool of *BRCA*-wildtype HGSOC could increase the proportion of PARPi-eligible patients.

## Targeting HR Function as a Chemosensitization Strategy

In addition to identifying cancers with inherent HR defects, the active modulation of HR capacity in BRCA-proficient cancers via targeting of BRCA function is an attractive therapeutic concept. Quinn et al. showed that downregulation of *BRCA1* by RNAi increased sensitivity to cisplatin in ovarian cancer cell lines (44), thus providing a rationale for the use of pharmacological agents in order to inhibit BRCA function. Several recent publications suggest that pharmacological targeting of BRCA1 and BRCA2 can sensitize *BRCA*-wildtype cancers to platinum-based chemotherapy and PARP inhibition. In the following sections, we will outline potential therapeutic strategies that target BRCA loss-of-function as a result of transcriptional downregulation or inhibition of protein activity. We will describe the molecular pathways regulating *BRCA* gene expression and their activation in HGSOC, and the transcription factors that mediate transcriptional activation of *BRCA1* and *BRCA2*. Finally, we will discuss different classes of targeted compounds for their potential use as chemosensitizing agents in BRCA-proficient HGSOC. We hypothesize that molecular targeting of HR function can reverse platinum resistance in HGSOC.

### Regulation of *BRCA1* and *BRCA2*

Control of BRCA1 and BRCA2 activity involves transcriptional regulation and post-translational modifications of the BRCA proteins. As part of the DDR, BRCA1 is phosphorylated by CHEK2 and ATM in normal cells and cancer cells following irradiation or exposure to alkylating agents [reviewed in Ref. (24)]. While DDR inhibitors may be able to sensitize cells to DNA-damaging agents, this article focuses on targeting genes and pathways that are activated specifically in cancer cells and required for *BRCA* gene expression and/or activity. Importantly, many oncogenic drivers and their downstream mediators, such as proliferation-associated transcription factors, are positive regulators of *BRCA1* and *BRCA2*. Activation of BRCA1 and BRCA2 by oncogenes offers the opportunity to selectively sensitize cancer cells to platinum by targeting defined genetic alterations that are not present in normal cells. Inhibition of oncogenic drivers may result in downregulation of *BRCA* mRNA and/or inactivation of BRCA proteins.

BRCA1 and BRCA2 are regulated in a cell cycle-dependent manner. In cultured cells, *BRCA1* and *BRCA2* mRNA expression is low under conditions of serum starvation, confluency, or other factors that induce G_0_ cell cycle arrest (45, 46). In contrast, rapidly proliferating cells express high levels of both *BRCA1* and *BRCA2* mRNA. This is in line with the documented function of BRCA1 and BRCA2 in HR, which occurs during the S and G_2_ phases of the cell cycle, and ensures DNA replication fidelity.

#### Transcriptional regulation of *BRCA1* and *BRCA2* by ETS, MYC, and E2F

**Figure 1:**
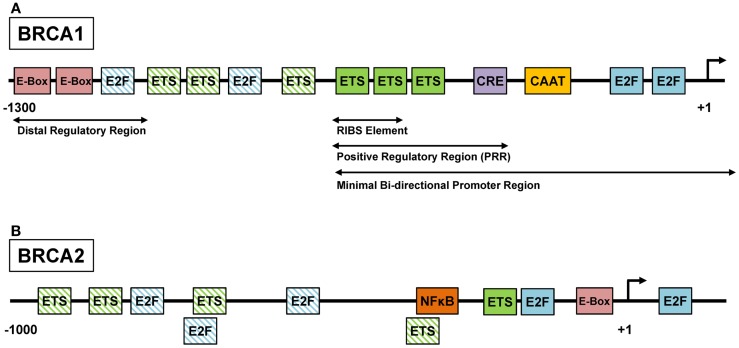
**ETS, MYC, and E2F regulate the *BRCA1* and *BRCA2* promoters**. **(A)** The regulation of the *BRCA1* promoter is complex and several regulatory sites have been identified. The positive regulatory region (PPR) located at the 5′ end of the promoter has been shown to be necessary and sufficient for *BRCA1* transcription, and contains sites such as the cyclic-AMP response element (CRE). Within the PPR, the three consecutive ETS factor binding sites are known as the RIBS element and have been shown to be bound by GA-binding protein α/β (GABPα/β), an ETS factor family member. **(B)** The *BRCA2* promoter contains several conserved recognition motifs for transcription factors including E-box, E2F, and ETS (49). *BRCA2* gene transcription may be activated by the binding of ELF1, an ETS factor, to the ETS recognition motifs or by the binding of USF1 and USF2, basic helix-loop-helix leucine zipper family members, to the E-box. Additionally, NF-κB can also bind the *BRCA2* promoter and induce genes expression (59). Functionally characterized binding sites are depicted by solid colored boxes. Putative binding sites are depicted by patterned boxes and were identified using the MatInspector software (Genomatix, Munich). Diagrams are not drawn to scale.

Several classes of transcription factors are involved in cell cycle progression and have been shown to regulate *BRCA1* and *BRCA2* expression. Initial analysis of the human *BRCA1* promoter identified a core promoter region that extends from about 250 bp upstream of the transcription start site (TSS) into the first exon (47, 48). This <300 bp region, which was associated with the highest promoter activity in reporter assays, contains several E2F and ETS binding sites as well as a CREB binding site (Figure 1A). The human *BRCA2* promoter has been characterized to a lesser extent but also contains functionally relevant E2F and ETS binding sites within its proximal region (Figure 1B) (49). Traditionally, E2F transcription factors are considered as the main mediators of G_1_–S progression. Activator E2Fs (E2F1–3) transcribe many of the genes involved in DNA replication, checkpoint control, and DNA repair (50). Importantly, E2F function couples proliferation and DNA repair by coordinating the induction of genes required for DNA synthesis, such as thymidine kinase (*TK1*) and dihydrofolatereductase (*DHFR*), and DNA repair, such as *BRCA1, BRCA2*, and *RAD51* (51). However, mouse models have demonstrated that while activator E2Fs are critical for cell cycle progression in some cell systems, E2F-independent proliferation occurs in others (52–54). There is mounting evidence that other classes of transcription factors can compensate for loss of E2F function in some cell types. For example, E2f1–3-null mouse retinal progenitor cells continued to divide possibly due to compensation by Mycn, a member of the MYC family of basic helix-loop-helix transcription factors (52). In breast cancer cells, MYC was found to directly regulate the *BRCA1* promoter via binding to distal regulatory regions (55) (Figure 1A). In addition, oncogenic ETS family transcription factors were shown to induce a subset of E2F target genes (56, 57), including *BRCA2* (58). Thus, a number of cancer-relevant transcription factors regulate *BRCA1* and *BRCA2*. In order to achieve effective downregulation of *BRCA* transcription, it is necessary to identify and target the main drivers of *BRCA* gene expression in different subgroups of HGSOC.

#### Pharmacologic targeting of oncogenic transcription factors driving *BRCA* expression

Some of the transcription factors driving *BRCA* gene expression are known oncogenes (*MYC*) or putative oncogenes in ovarian cancer, based on functional data and evidence of genetic activation in primary HGSOC: *MYC* is amplified in 30% of HGSOC (6), and *E2F3* is amplified in about 10% (60). While most transcription factors are not easily druggable with currently available agents, targeting of BET bromodomain proteins has been described as an effective means of inhibiting MYC-dependent transcription (61). Preclinical studies with the small molecule inhibitor JQ1 have yielded promising results in MYC-dependent hematologic malignancies, medulloblastoma (62), and *KRAS*-mutant lung cancer (63), but its compatibility with platinum-based chemotherapy, as well as its effect on *BRCA* gene expression, has yet to be established.

ETS family transcription factors have been implicated in platinum resistance. ETS1 was shown to be overexpressed in C13 cells, a cisplatin-resistant derivate of 2008 ovarian cancer cells (64), and ectopic expression of ETS1 in 2008 cells conferred platinum resistance. Similarly, ETV4 (PEA3) is overexpressed in cisplatin-resistant PEO1 ovarian cancer cells (65). Both the *BRCA1* and the *BRCA2* promoter are bound and activated by ETS transcription factors: GA-binding protein α/β (GABPα/β) binds the RIBS element in the *BRCA1* core promoter (Figure 1A), and overexpression of GABPα/β in breast cancer cells was able to stimulate *BRCA1* promoter activation (66). Similarly, *BRCA2* gene transcription may be activated by the binding of ELF1, another ETS family member (49) (Figure 1B).

Studies in prostate cancer suggest a targeting strategy for oncogenic ETS factors. Constitutive activation of ERG, ETV4, or ETV5 following gene fusion with the *TMPRSS2* promoter renders the fusion gene oncogenic in prostate cancer cells (67). ERG was shown to interact with PARP and require PARP activity for its transcriptional activity. Inhibition of PARP by Olaparib specifically sensitized ERG-driven prostate cancer xenograft to the alkylating agent, temozolomide (68). Similarly, ETS-dependent, BRCA-proficient ovarian cancers may be susceptible to PARPi. However, ETS gene fusions have not been detected in ovarian cancer, and biomarkers of ETS dependency have yet to be identified in HGSOC. Thus, direct targeting of transcription factors is an interesting therapeutic strategy, but requires additional studies prior to translation into the clinic.

### Regulation of *BRCA1* and *BRCA2* by oncogenic signaling

**Figure 2:**
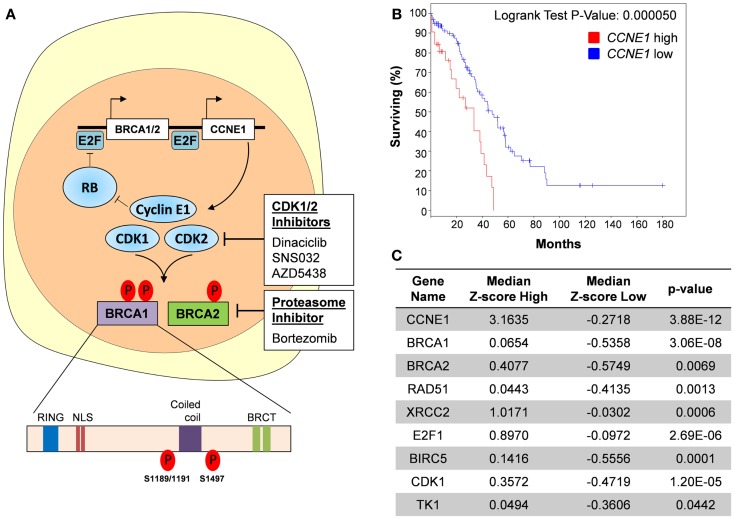
**Targeting cyclin E1-dependent ovarian cancers**. **(A)** Cyclin E1 function is dependent on it interaction with CDK1 and CKD2. CDK1/2 inhibitors inhibit this interaction and cause E2F-mediated transcriptional downregulation of *BRCA1* and *BRCA2*. CDK1/2 inhibition may also prevent their phosphorylation and subsequent activation of the BRCA1 and BRCA2 proteins. Alternatively, use of Bortezomib, a proteasomal inhibitor, can inhibit HR function and potentially sensitize cyclin E1-dependent tumors to platinum-based chemotherapy. NLS, nuclear localization signal; BRCT, BRCA1 C-terminus domain. **(B)** Kaplan–Meier curve showing that *CCNE1* overexpression (*n* = 32 tumors, *Z*-score ≥ 2) in HGSOC was associated with significantly reduced overall survival (OS) as compared with tumors with normal or low expression of *CCNE1* (*n* = 97 tumors, *Z*-score < 2). Median OS for cancers with *CCNE1* high and normal/low expression were 33.44 and 47.47 months, respectively. A subset of 129 HGSOC tumors from the TCGA dataset was used for this analysis and was selected based on p53-mutant and *BRCA1-* and *BRCA2-*wildtype status as well as availability of RNAseq V2 data. Data were accessed using the cBioPortal for Cancer Genomics maintained by the Computational Biology Center at Memorial Sloan-Kettering Cancer Center. **(C)** Increased expression of HR and DNA repair-related genes in cyclin E1-overexpressing tumors. Known E2F targets, *E2F1, BIRC5* (survivin), *CDK1* (CDC2), and *TK1* are included for reference. Median *Z*-score values for each *CCNE1* expression subset are shown; *P* values were determined using Student’s *t*-test.

**Figure 3:**
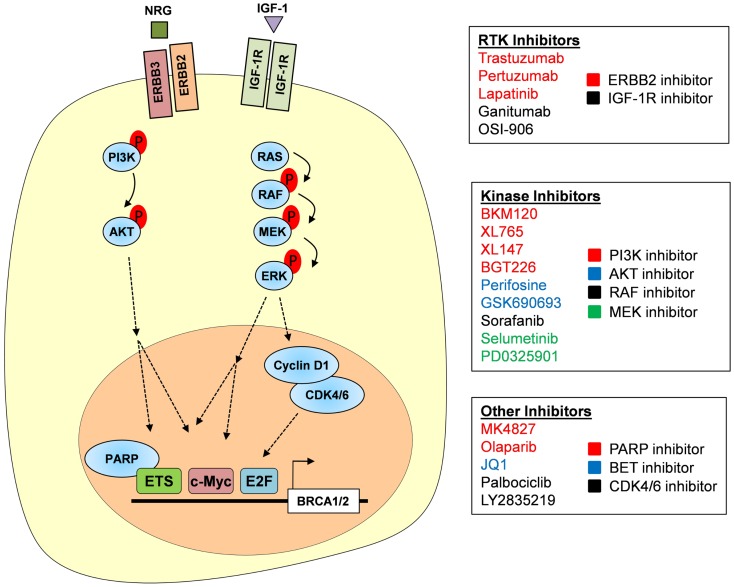
**Targeting the RTK–PI3K/MAPK axis**. RTKs, including ERBB2/ERBB3 and IGF-1R, are activated by their respective growth signals and are capable of signaling through both the PI3K–AKT and MAPK pathways. Subsequent activation of AKT and ERK allows for the phosphorylation of multiple transcription factors including ETS and MYC that are important in *BRCA1* and *BRCA2* gene transcription. Activation of ERK also induces cyclin D1 production, which in complex with CDK4/6 promotes E2F-mediated gene transcription. We propose that *BRCA1* and *BRCA2* expression could be inhibited at each of these levels; inhibitors of RTKs, PI3K signaling, MAPK signaling, and CDK4/6 are listed. PARPi, such as Olaparib, may also downregulate *BRCA* gene expression as PARP1 is required as a co-factor for some ETS family members.

A more immediate option may present itself in targeting the signaling pathways that lead to activation of transcription factors. ETS, MYC, and E2F transcription factors are downstream mediators of several oncogenic signaling pathways (Figures 2A and 3). Inhibition of these pathways may result in loss of transcriptional activity and subsequent downregulation of *BRCA* gene expression. TCGA identified the retinoblastoma (RB) pathway, phosphatidylinositide 3-kinase (PI3K), and RAS signaling as the most frequently altered signaling pathways in HGSOC (6).

#### The RB pathway

The RB pathway governs G_1_/S transition in mammalian cells. In proliferating cells, growth factor or oncogene-induced expression of cyclin D results in activation of the cyclin-dependent kinases, CDK4/6, and inactivation of the RB protein. RB forms complexes with E2F1–3 proteins and inhibits their transactivating activity. Phosphorylation of RB by CDK/cyclin complexes unleashes E2F activity, resulting in expression of E2F target genes, including cyclin E1 (*CCNE1*), *CDK2*, and *E2F1–3*, which amplify the signal in a positive feedback loop and transition the cell from G_1_ to S phase. The CDK/cyclin–RB–E2F axis (RB pathway) is frequently deregulated in cancer as a result of mutations or deletions in *RB1* (10% in HGSOC), amplification of cyclin genes (*CCNE1* is amplified in 20% of HGSOC, *CCND1* is amplified in 4%), or functional loss of endogenous CDK inhibitors (CDKi), such as p16^INK4A^ (*CDKN2A*, downregulated in 30% of HGSOC) (6). The net result of RB pathway alterations in cancer is increased proliferation and elevated E2F activity compared to normal tissue. Wang et al. studied regulation of the murine *Brca1* promoter by the CDK/cyclin–RB–E2F axis. Using a luciferase *Brca1* promoter construct (+6 to −1003), ectopic cyclin D1 induced luciferase activity while RB suppressed activity. They also identified a conserved 5′-*GCGGGAAT*-3′ E2F binding site at −37 to −19 relative to the TSS (−23 to −5 in the human *BRCA1* promoter) and demonstrated physical binding of E2F protein to this region (69) within the *BRCA1* core promoter (Figure 1A). Hence, the RB pathway directly controls *BRCA* expression via regulation of E2F activity, and targeting of cancer-specific RB pathway lesions, such as *CCNE1*, may result in downregulation of *BRCA* gene expression.

### Targeting *CCNE1* dependency

#### Direct targeting of CDK/cyclin signaling

*CCNE1*-amplified cancers make up a large subgroup (20%) of HGSOC that lack *BRCA* mutations (6) and are therefore less likely to respond to PARPi. For likely the same reason, *CCNE1*-amplified tumors were reported to be among the most chemoresistant HGSOC (70), and recurrent *CCNE1*-amplified HGSOC continue to be dependent on the presence of the *CCNE1* amplicon (71). The *CCNE1* gene product, cyclin E1, is a co-factor for cyclin-dependent kinases 1 and 2 (CDK1/2) and activates *BRCA1* and *BRCA2* transcription via E2F transcription factors (50) (Figure 2A). Therefore, *CCNE1*-amplified HGSOC often express high levels of wildtype *BRCA1* and exhibit an “anti-BRCAness” phenotype in terms of their relative resistance to platinum chemotherapy. Using the TCGA dataset, we show that cyclin E1-overexpressing, *BRCA*-wildtype HGSOC have significantly reduced OS compared to *BRCA*-wildtype cancers with lower cyclin E1 expression (Figure 2B). This suggests that high levels of cyclin E1 confer an added advantage to BRCA-proficient cancers. We attribute this, at least in part, to increased expression of several HR genes, including *BRCA1, BRCA2, RAD51*, all of which have significantly higher RNA levels in cyclin E1-overexpressing cells (Figure 2C).

CDK inhibitors may be a therapeutic option for cyclin E1-dependent HGSOC, including *CCNE1*-amplified cancers. Cyclin E1 has the highest affinity for CDK2, its main binding partner in actively cycling cells (72–74), and CDK1 (CDC2) (75). Both CDK2 and CDK1 directly phosphorylate BRCA1 (76, 77) (Figure 2A). Furthermore, a functional link between CDK1 and BRCA1 has been established in lung cancer. Using an inducible shRNA targeting CDK1 in a human non-small cell lung cancer cell line, Johnson et al. showed that CDK1 contributes to S phase checkpoint control following DNA damage (77). CDK1 directly phosphorylated BRCA1 at several serine residues and is required for the formation of BRCA1 foci following DNA damage. Genetic depletion or pharmacological inhibition of CDK1 sensitized BRCA1-proficient cancer cell lines to cisplatin (77) and PARPi (78). Similarly, BRCA2 is phosphorylated by both CDK1 and CDK2 (79).

While there are still no selective inhibitors of CDK1 or CDK2, latest generation CDKi have increased specificity and potency compared to early CDKi, such as flavopiridol, which failed in the clinic. The compound Dinaciclib inhibits CDK1/2/5/9 at low nanomolar concentrations (IC_50_ values: CDK1: 3 nM, CDK2, 1 nM, CDK5: 1 nM, CDK9: 4 nM). It induces apoptosis in model systems and prevents tumor progression in A2780 ovarian cancer xenografts (80, 81). We hypothesize that of the currently available CDKi, Dinaciclib may have the best therapeutic potential in cyclin E1-dependent ovarian cancer. Our unpublished data show that Dinaciclib exposure results in downregulation of *BRCA1* and *BRCA2* and sensitized cyclin E1-dependent ovarian cancer cells to cisplatin. Hence, Dinaciclib may have a dual effect on BRCA1 and BRCA2 by causing E2F-mediated transcriptional downregulation and inhibiting CDK1/2-mediated activation of the BRCA proteins (Figure 2A).

Dinaciclib is currently being evaluated in a phase 3 clinical trial for chronic lymphocytic leukemia (CLL) and may have clinical potential in cyclin E1-dependent HGSOC as a chemosensitizing agent. In order to select eligible patients, *CCNE1*-amplified cancers can be easily identified by fluorescence *in situ* hybridization (FISH). Other available CDK2 inhibitors, including Roscovitine, SNS032 (82), and AZD5438 (83), may have similar chemosensitizing potential. CDK1, the only mammalian CDK required for proliferation in all cell types (84), is not inhibited by SNS032, rendering this compound potentially less potent but also less toxic to normal cells. In principle, other CDKi such as the CDK4/6 inhibitor Palbociclib (formerly PD0332991) could be used to reduce *BRCA* expression in cyclin D-dependent HGSOC. However, Palbociclib was shown to be cytostatic in most systems (85–87) and may thus interfere with the cytotoxic activity of platinum agents.

#### Targeting of HR components induces synthetic lethality in *CCNE1*-amplified HGSOC

An independent approach to target *CCNE1*-amplified HGSOC identified the proteasome inhibitor, Bortezomib (88). In contrast to CDKi, Bortezomib primarily affects the homologous repair system itself, resulting in synthetic lethality in *CCNE1*-amplified cancer cells: a genome-wide shRNA screen in 102 cancer cell lines revealed that BRCA1 was specifically required in *CCNE1*-amplified cell lines. Genetic depletion of *BRCA1* resulted in significant loss of viability in *CCNE1*-amplified cells, including the ovarian cancer cell line OVCAR3, whereas a lesser effect was observed in *CCNE1*-wildtype cells, such as the SKOV3 cell line (88). This suggests a synthetic lethal relationship between *CCNE1* amplification and loss of BRCA1 function and provides a potential explanation for the observed mutual exclusivity between *CCNE1* amplification and *BRCA* mutation (6, 89). In addition to *BRCA1*, the shRNA screen identified the DNA repair genes *ATR* and *XRCC2* as specific genetic hits in *CCNE1*-amplified cell lines, suggesting that inhibition of the DDR may be a therapeutic strategy in *CCNE1*-amplified ovarian cancer. To this end, the authors tested Bortezomib, a potent inhibitor of the HR system, in a panel of ovarian cancer cell lines and found that *CCNE1*-amplified lines were most sensitive. While Bortezomib had minimal activity as a single agent in recurrent ovarian cancer (90) combination with platinum resulted in clinical activity (91). As discussed for CDKi, specific selection of patients with *CCNE1*-amplified cancers for Bortezomib treatment may further increase the response rate.

### Receptor tyrosine kinase, PI3K, and RAS signaling

In addition to genetic aberrations within the RB pathway, several oncogenic signals contribute to cell cycle deregulation and E2F activity. Oncogenic *KRAS* (amplified in 11% of HGSOC), MYC, and receptor tyrosine kinases (RTK) all converge on cyclin D and require its function for their oncogenic activity (92–94). Moreover, a synthetic lethal relationship was described for KRAS and CDK4, the binding partner of cyclin D (94). KRAS and ERBB2 signal through the mitogen-activated kinase (MAPK) signaling pathway, which culminates in activation of ERK (Figure 3). Phosphorylation by ERK activates multiple cellular target proteins, including ETS, CREB, and MYC. The molecular link between oncogenic signaling pathways and expression of *BRCA1* and *BRCA2* offers additional opportunities for therapeutic intervention, including direct targeting of ERBB2 and other RTK, targeting of the MAPK pathway by specific MEK or ERK inhibitors, or targeting of the PI3K–AKT axis by specific inhibitors (Figure 3). However, due to the complex signaling events elicited by RTK and other oncogenic pathways, regulation of *BRCA* gene expression is only one of many downstream events, some of which may actually interfere with the intended chemosensitizing effect. Functional studies are needed to determine the compatibility of individual targeted agents with platinum and PARPi.

#### Targeting RTK signaling and downstream pathways

Although amplifications or mutations of individual RTK are rare events in primary HGSOC (*ERBB2* amplification: 3%, *ERBB3* amplification: <5%, *IGF1R* amplification: <4%, Figure 3), altered ERBB receptor signaling and overexpression of the downstream RTK mediator ETV4 were found in cisplatin-resistant PEO1 cells (65). RTK can be targeted directly by small molecule inhibitors (e.g., Lapatinib for ERBB2) or antagonistic antibodies (e.g., Trastuzumab or Pertuzumab for ERBB2). However in unselected patients, the addition of Pertuzumab to carboplatin-based chemotherapy did not result in prolonged progression-free survival (95), highlighting the importance of patient selection and companion diagnostics to identify likely responders to RTK inhibitors. Activation of the MAPK and PI3K–AKT signaling pathways by RTK, or as a result of amplification, offers additional potential therapeutic targets [also reviewed in Ref. (96)]. Both *PIK3CA*, the gene encoding the catalytic subunit of PI3K, and the *AKT* genes are frequently amplified in HGSOC (*PIK3CA*: 28%, *AKT1*: 5%, *AKT2*: 8%, *AKT3*: 9%), indicating their oncogenic roles in ovarian cancer. Interestingly, AKT was shown to directly phosphorylate BRCA1 at serine 694 and promote its protein stability (97). A second AKT phosphorylation site at threonine 509 was described but its functional implications are unknown (98).

Functionally, a recent study in triple negative breast cancer demonstrated that PI3K signaling was required for BRCA1 function. Ibrahim et al. showed that inhibition of PI3K phenocopied loss of BRCA1 and induced synthetic lethality in combination with Olaparib (99). Loss of PI3K resulted in reduced *BRCA1* expression both in cell line models and in patient-derived tumor xenografts. Combined treatment with BKM120, a small molecule PI3K inhibitor, and Olaparib significantly delayed tumor progression in two out of three xenografts whereas single agent treatment had little effect on tumor progression. On the molecular level, the MAPK pathway mediated the transcriptional effect on *BRCA1* via ETS1-dependent downregulation of *BRCA1* (99). Interestingly, BKM120 treatment resulted in activation of the MAPK pathway and phosphorylation of ETS1 by ERK, indicating a repressor function for ETS1 in this system. This finding highlights the complexity of *BRCA* gene regulation and its dependency on genetic context. Due to feedback mechanisms and functional compensation among RTKs, downstream kinases, and transcription factors, specific pharmacological combinations may be limited in their effectiveness to small genetically defined subsets of HGSOC. Moreover, as a result of the genetic complexity and genomic instability that is a hallmark of HGSOC, individual cancers are likely to harbor or develop resistant cell clones to any given combination. Thus, it will be important to identify multiple agents targeting different pathways, all of which should be characterized with respect to: (1) their ability to induce BRCA loss-of-function, (2) their compatibility with platinum and/or PARPi, both in terms of toxicity and mechanism of action, and (3) accompanying biomarkers that allow for careful patient selection.

### Targeting BRCA protein stability

A different approach to inhibit BRCA1 function involves targeting of the chaperone protein heat shock protein 90 (HSP90), required for BRCA1 protein stability (100). Interestingly, *HSP90AB1* is also amplified in a small subset of HGSOC (6). Specific inhibition of HSP90 in BRCA1-proficient breast cancer cells-induced BRCA1 ubiquitination and proteasomal degradation ultimately resulting in compromised DSB repair by HR. HSP90 inhibition sensitized BRCA1-proficient breast and ovarian cancer cells *in vitro* to carboplatin treatment at concentrations similar to BRCA1-mutant cells (100). In a subsequent study, treatment with the pan-histone deacetylase complex (HDAC) inhibitors vorinostat or panobinostat induced hyperacetylation of HSP90 gene (*HSP90AA1I0*), thereby inhibiting its chaperone function and leading to proteasomal degradation and depletion of BRCA1 (101). Of clinical significance, treatment of human triple negative breast cancer cell lines with vorinostat was able to induce BRCA1 degradation and a subsequent BRCAness phenotype, which synergistically induced cell death in combination with PARPi or cisplatin (102). Based on these preliminary studies, it is suggested that treatment of platinum-resistant HGSOC with HDAC inhibitors may also be able to induce a BRCAness phenotype and resensitize these cells to platinum or other targeted agents. Trials evaluating vorinostat as a single agent in recurrent epithelial ovarian cancer showed it had limited activity (103). Vorinostat may show better therapeutic efficacy as a biologic response modifier in combination with platinum-based chemotherapy and trials assessing this use in ovarian cancer are underway.

## Concluding Remarks

In HGSOC cells, a functional HR system and intact BRCA1 and BRCA2 function are often associated with resistance to platinum-based chemotherapeutic agents and PARPi. A plethora of targeted agents are currently available to modulate BRCA function via transcriptional or post-translational intervention. With the advent of novel diagnostic tools, such as the use of deep sequencing to repeatedly profile cancers throughout their evolution, it should become possible to predict rational therapeutic combinations that prevent or at least delay the onset of platinum resistance and should ultimately result in improvements in OS.

## Conflict of Interest Statement

The authors declare that the research was conducted in the absence of any commercial or financial relationships that could be construed as a potential conflict of interest.
